# Supplementary material for: Conceptualization and establishment of value-based healthcare in Saudi Arabia: A scoping review
Source: J Taibah Univ Med Sci. 2024 Aug 6;19(4):835–46. doi: 10.1016/j.jtumed.2024.07.008 (PMC11369466; doi:10.1016/j.jtumed.2024.07.008)
Supplement: Multimedia component 1 [file mmc1.docx]

**Title**

Conceptualization and Establishment of Value-Based Healthcare in Saudi Arabia: A Scoping Review

**Abstract**

**Objectives:** Value-based healthcare (VBHC) represents a paradigm shift in healthcare delivery through optimizing patient outcomes relative to the costs of achieving those outcomes. This scoping review is aimed at revealing critical insights into the conceptualization and establishment of VBHC in the context of Saudi Arabia, a nation in a critical stage of healthcare transformation.

**Methods:** A scoping review was conducted by using online databases and official websites with a timeframe of 2017–2023. This review included 14 pieces of literature, comprising six research articles, six government documents, and two reports.

**Results:** The findings highlight increasing alignment with the definition of global VBHC principles, notably the emphasis on patient outcomes as a primary metric of healthcare value. Furthermore, financial reform has signaled a real move toward VBHC in the Kingdom, through a gradual shift from volume-based payments to value-based payments. However, the diverse interpretations and applications of VBHC across the examined literature indicate a promising stage of implementation characterized by evolving definitions and practices tailored to local needs and constraints.

**Conclusion:** This scoping review describes the current landscape of VBHC conceptualization and establishment, highlighting the substantial progress achieved and the future challenges.

**Keywords:** Conceptualization; Health Sector Transformation Program; Saudi Arabia; Utilization; Value-based healthcare.

**Word Count:** 4,105

**Introduction**

Saudi Arabia’s healthcare system is experiencing growing pressure from chronic diseases, aging populations, and increasing health expenditures (1). Consequently, optimizing health system access and efficiency has been central to health transformation. In alignment with Vision 2030, the Saudi Ministry of Health (MoH) has introduced a national health transformation program driven by a value-based healthcare (VBHC) system. The opening statement of the transformation program states the following: “The health sector transformation program depends on the principle of value-based care, which ensures transparency and financial sustainability by promoting public health and preventing diseases, in addition to applying the new model of care related to disease prevention” (2).
This transformation represents a departure from traditional volume-based healthcare models to a VBHC system, by prioritizing outcomes over volume, embracing a patient-centered approach, and emphasizing cost-effectiveness. Unlike traditional models that incentivize quantity of services, VBHC aligns incentives with patient outcomes, fosters data-driven decision-making, and promotes coordinated care across the continuum. By focusing on preventive care, population health management, and efficient use of resources, VBHC is aimed at improving patient satisfaction, enhancing quality of care, and controlling costs (3–5). This transformation will require tremendous changes to the infrastructure of the Saudi healthcare system.

The basic definition of value, as introduced by Porter and Teisberg (2006), is “patient outcome achieved per dollar spent”(6). With the emergence of differing practices and applications of VBHC, the global definitions of value in healthcare place conflicting emphasis on cost, cost-effectiveness, and better health outcomes; however, across these definitions, a key element is delivering the outcomes that patients require (7,8). Thus, in the general understanding of the concept of VBHC, health resources are used efficiently to deliver the right and necessary care at the right time (9).

In the Saudi context, as part of the MoH’s commitment to move toward a VBHC system, the Value in Health (VIH) center was established to improve value in the healthcare sector by building capabilities and sharing knowledge (10). However, because VBHC was only recently introduced in Saudi Arabia, the current literature lacks an overview of the conceptualization and level of establishment of VBHC in Saudi Arabia, and a comprehensive exploration of these aspects is necessary (11).

Consequently, in response to the status of VBHC in Saudi Arabia, this study asked how the conceptualization and establishment of VBHC in Saudi Arabia is portrayed in the literature.

The objective of this review was to a) understand the conceptualization of the VBHC strategy, as documented in the Saudi literature, and b) to identify the extent of establishment of the VBHC strategy in Saudi Arabia.

The conceptualization of VBHC pertains to how the authors of a given study define the term, whereas VBHC establishment involves activities performed by the concerned national entities to accelerate VBHC implementation. The level of establishment was evaluated with respect to the comprehensive VIH definition published by the VIH center, which defines VBHC within the Saudi context as follows: “improving value in Health is achieving the best health outcomes with the optimal and fair allocation and best utilization of resources where ‘outcomes’ relate to benefits delivered for individuals, communities and the population; and, ‘resources’ include all human, capital and natural resources”(12). This definition provides a robust framework for assessing the extent to which the VBHC concept has been established in the Saudi healthcare sector.

The VIH framework (Figure 1) presents the key value components in the Saudi health system, wherein outcomes represent measurement of clinical outcomes at the following three levels of any society: populations, communities, and individuals. Resources, in contrast, are grouped into allocation of resources that are assessed through financial reforms and utilization of resources that are indicated in provider reforms (13).


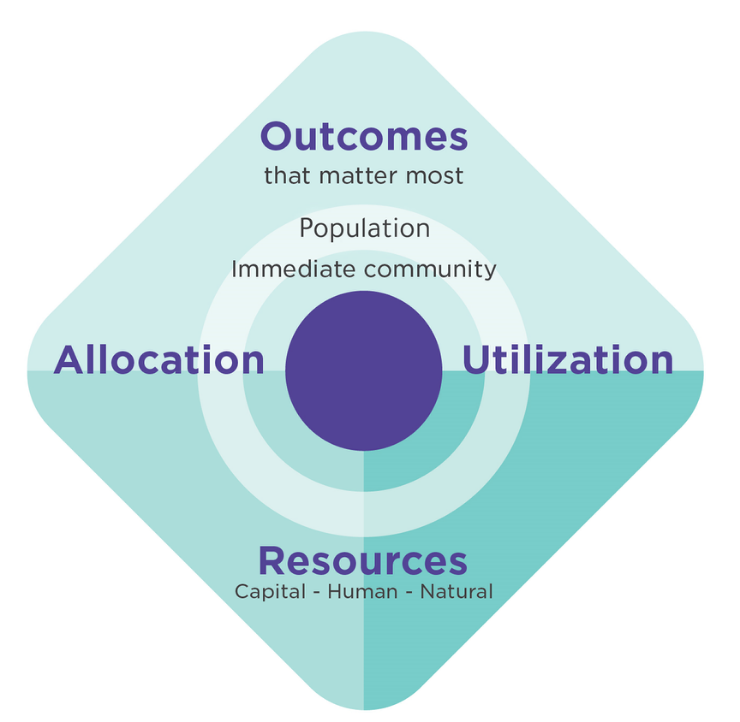


Figure 1: Value in Health Framework (13)

**Methods**

Study Design

Given the limited literature on VBHC in Saudi Arabia, and the broad research question of this review, a scoping review was deemed more appropriate than other types. As stated by Munn et al., scoping reviews “are useful for examining emerging evidence when it is still unclear what other, more specific questions can be posed” (14). Thus, this approach enabled a broad explanation of VBHC in the Saudi context.

The scoping review was conducted in compliance with the Joanna Briggs Institute guidelines and the Arksey and O’Malley Framework (15). The Preferred Reporting Items for Systematic Reviews and Meta-Analyses extension for Scoping Reviews (PRISMA-ScR) was followed (16). Moreover, the PRISMA-ScR checklist was used to validate that all necessary reporting items were addressed (Appendix A).

Search Strategy

PubMed, Scopus, and Web of Science were strategically selected as primary databases for this scoping review to ensure comprehensive coverage of both global and region-specific literature on VBHC in Saudi Arabia. These databases were identified as optimal sources because of their extensive indexing of medical and health science literature, including a wide range of peer-reviewed articles, reports, and governmental documents relevant to VBHC concepts and implementation. The choice of databases was further supported by their known inclusion of studies addressing health system transformations and policy implications, which are critical for understanding the VBHC landscape in the context of Saudi Arabia's health sector reforms. This approach aligned with the scoping review's objective to capture a broad spectrum of evidence on VBHC conceptualization and establishment, to facilitate a thorough exploration of the subject matter within the specified timeframe starting from 2017. The syntax for each database was created, and keywords were included, as shown in Table 1.

Further searches of relevant government and VBHC-related agency websites were undertaken, including those of the MoH, VIH center, Center for National Health Insurance, Saudi Health Council, PHA, Council of Health Insurance (CHI), Saudi Commission for Health Specialties, and Global Innovation Hub for Improving Value In Health. The websites were chosen according to the key organizations within the Saudi health sector dedicated to implementing VBHC.

Study Eligibility

The inclusion criterion was full-text articles reporting evidence focused on VBHC in Saudi Arabia. No restriction was placed on article type, because of the scarcity of articles, governmental documents, and reports published in English between January 2017 and September 2023. Backward and forward citation searches were conducted through the citationchaser website (17).

The exclusion criteria were articles not published in English, articles with a scope of evidence not including VBHC, articles published before 2017, and articles without full-text availability.

Study Selection

The search results were imported into Zotero after duplicates were removed. Eligibility screening was conducted with the online platform SR-accelerator (18). First, titles and abstracts were screened by two reviewers (B.H. and A.A.), and disagreements regarding the articles were resolved through discussion between reviewers. If a consensus was not reached, a third reviewer made the final decision (F.A.). The article selection process was documented according to PRISMA-ScR, as illustrated in the study flowchart (Figure 2).

Records identified through database search (n=43)

PubMed = 13

Scopus = 17

Web of Science = 13

Official website documents = (n=8)

Records removed before screening:

Duplicate records removed (n=32)

Records screened (n=20)

Official websites = (n=8)

Records excluded by title and abstract screening (n=6)

Records assessed for eligibility (n=14)

Official websites = (n=8)

Records excluded (8)

- Full text not available (n=3)
- Scope not VBHC related (n=5)

Included studies (n=6)

Official websites = (n=8)

Identification

Screening

Included

Figure 2: PRISMA-ScR flowchart

Data Extraction and Synthesis (Data Charting)

Data extraction was performed by two reviewers (B.H. and A. A.). The following extraction fields were used to organize and summarize the findings: authors, year, study type, aim, VBHC conceptualization (definition), VBHC level of establishment (outcome: individuals, communities, and population; resources: allocation and utilization) and the concerned entities.

Subsequently, the findings were reorganized to address the review objectives. Data in the field of VBHC conceptualization were categorized to indicate how VBHC is conceptualized in the current Saudi literature (review objective 1). Data from the VBHC level of establishment and concerned entities were used to identify progress in VBHC in Saudi Arabia (review objective 2).

**Results**

This review included 14 pieces of literature, comprising six research articles, six government documents and two reports, which were comprehensively screened and reviewed to allocate data to one of the two review objectives. Article type is described in the evidence information column in Table 2, and the entities originating these documents are described in the evidence information column in Table 3.

VBHC Conceptualization

Of the six research articles examined, two provided (19,20) a precise definition of VBHC drawn from Porter's original concept (6). In the remaining articles, the VBHC concept was articulated solely by referencing its elements or objectives, without provision of a specific definition of the VBHC concept (21–24). Each article focused on specific elements of the VBHC definition. For example, Alkhudair and colleagues focused on the implementation of value-based agreements between payers and pharmaceutical companies; therefore, their explanation of VBHC focused on cost, stating that VBHC is aimed at "balancing the clinical benefits versus costs when making decisions related to drug reimbursement” (21). Similarly, when the term "value" was described by Memish and colleagues, it was associated exclusively with cost (22). In contrast, two other articles highlighted the crucial aspect of outcome measurement, while identifying and prioritizing the elements valued most by patients (23,24). An overview of the definitions is presented in Table 2.

The six governmental documents included two documents published by the Health Sector Transformation Program (HSTP) under the MoH. In one document, VBHC was defined as "outcome divided by cost" (25), drawing from Porter’s VBHC definition (6). The second document, despite lacking a precise definition of VBHC, outlined its key elements, which encompassed patient-centeredness, cost control, and incentivization of health providers (26).

The Saudi VIH center published three documents, two of which stated the definition published by the VIH, which was tailored to the Saudi context with consideration of the global definitions (12,27). The difference in this definition was in use of the term "resources" instead of "cost" to convey a broader and more flexible interpretation encompassing monetary and non-monetary resources. However, the third document from the VIH center merely referenced the elements of VBHC in the narrative (28). Finally, the sixth document was a white paper from the CHI, which also defined VBHC according to the perspective of Porter and Teisberg (29).

The final evidence comprised two reports published by the Global Innovation Hub for Improving Value in Health. The first report, published in 2021, also defined VBHC according to the definition of Porter and Teisberg (11). In contrast, the other report focused more on the concept of high-value systems than VBHC (30). An overview of the definitions of governments document and reports is provided in Table 3.

VBHC Establishment

Regarding the VBHC level of establishment, evidence was organized according to the elements of the VIH framework: outcome, resource allocation, and utilization.

Three governmental documents and one report focused on the plan and roadmap to guide the transformation to VBHC in Saudi Arabia. Two were published by the HSTP, the first of which introduced the transformation to VBHC; therefore, the document focusing on how the Kingdom is planning to establish and implement this concept fully details the strategic plan for this transition, including provider reforms, financial reforms, governance development, and capacity building (25). The second document, from 2021, includes more focused details on the delivery plan for the entire sector and includes a section on the implementation of VBHC (26). The third document provides a comprehensive understanding of VBHC in the Saudi context and outlines the key components considered essential for VBHC strategy implementation in the healthcare sector (31). Furthermore, a report published by the Global Innovation Hub in 2021 underscores the essential role of this center in expediting knowledge transfer pertaining to VBHC (11).

The remaining ten pieces of literature were divided into two sets: five focused on establishing the outcome measurement, and the other five focused on reforms targeting resource allocation and utilization.

Outcome Measurement

Three articles focused on outcome measurement. The first article describes establishing the Patient Experience Measurement program, targeting improvements in patients experience at the national level, and the MoH Center of Patient Experience, in comparison with international practice (19). The second article describes the development of the National Health Performance framework under the Saudi Health Council, with an aim to unify health performance measurement in Saudi Arabia and to “provide further insights and visibility regarding the performance of the system as pertains to specific condition and, in turn, allow for the measurement of value at the level of specific condition” p. 301 (24).

The third article indicates that the involvement of physicians in outcome measurement has been limited. Notably, that research represented a first step toward implementing a disease-specific outcome set (23).

Moreover, two governmental documents published by the VIH center focused on initiatives and programs to establish proper measurement of patient experiences and patient outcomes (27). In 2020, the center collaborated in a multi-year research program with the Organisation for Economic Co-operation and Development (OECD) to assess patient-reported outcomes and experiences in primary healthcare, in an initiative known as the PaRIS project. The second document, released a year later, in 2021, delves deeper into the advancements of the PaRIS project, which is currently in the trial phase, involving tool validation and testing of measures on a sample. The primary goal of this phase is to assess patient-reported outcomes and experiences, to identify potential areas for enhancing care provision and working toward achieving improved value (28). Table 4 provides an overview of the establishment levels on outcome measurement.

Resource Allocation

Resource allocation pertains to financial reforms that compose a substantial portion of the visible initiatives in the Saudi health sector. Four pieces of literature outlined programs indicating a real move toward VBHC. The evidence revealed a gradual shift from volume-based payments to value-based payments, through the introduction of financial reforms aligned with value-based principles, such as bundled payments, accountable care organizations, value-based agreements, and capitation. These payment models encourage efficiency, coordination, and improved outcomes (20,21,29,30)

Elaborating on this element, Alkhudair and colleagues (21) have examined the elevated expenses associated with medication and have explored how the strategic implementation of VBHC in Saudi Arabia might enhance the management of these substantial costs. Their study reveals the current existence of value-based agreements in Saudi Arabia, which are integral components of the ongoing shift toward VBHC payment models. These agreements, involving reimbursement methods between payers and medical product manufacturers, are exemplified by the establishment of five agreements between the MoH and pharmaceutical companies in Saudi Arabia.

Abu-shraie and colleagues (20) have also shed light on the aspects of financial reforms aligned with the VBHC strategy. Risk-sharing agreements in the form of financial-based agreements have been introduced in the Saudi market between payers and medical product manufacturers “to allow for rapid access to innovative medication.” These financial-based agreements are increasingly becoming more frequently used than outcome-based agreements, both nationally and internationally.

An important aspect is the tangible progress made by the CHI on a national scale in formulating a strategy for implementing value-based payments (29). Key initiatives driving these reforms include implementation of the minimum dataset and the introduction of the National Platform for Health and Insurance Exchange Services (NPHIES), to facilitate the exchange in health information and insurance services. Table 5 provides an overview of the establishment levels in resource allocation.

Resource Utilization

Finally, resource utilization focuses on provider reforms limited to the development of 12 national clinical guidelines, as led by the National Guideline Center, which was established in collaboration with the Health Holding Company and the Saudi Health Council. These clinical guidelines will contribute to improving the understanding and engagement of healthcare providers in the delivery of value-based care by incorporating evidence-based practices, focusing on outcomes, and fostering a patient-centered approach (22). Table 6 provides an overview of the establishment levels in resource utilization.

**Discussion**

VBHC Conceptualization

For the first objective, evidence regarding the conceptualization of the VBHC was examined. Our review indicated a reliance predominantly on Porter's (6) definition among Saudi-based studies and policy documents, thus indicating a strong foundational understanding of VBHC principles. Nonetheless, each piece of literature presented a distinct expression of the concept associated with the scope of work. The emergence of a locally tailored definition by the Saudi VIH center underscores a critical move toward contextualizing VBHC to address specific challenges and opportunities within the Saudi healthcare system. This endeavor reflects a critical understanding that the successful implementation of VBHC requires not only a shift in healthcare delivery models but also a cultural shift among healthcare stakeholders toward a shared vision of value. Moreover, the center aimed to reach a consensus definition among local stakeholders; however, our review indicated that most of the literature has used a definition based on Porter’s definition rather than on Saudi value in the health sector. Notably, other countries implementing VBHC before Saudi Arabia have faced similar challenges in reaching a consensus definition among local stakeholders (32,33).

The variations in VBHC conceptualization, with some studies focusing on cost containment and others focusing on outcome optimization, mirrored global debates regarding balancing cost and quality in healthcare. This diversity indicated the use of a multifaceted approach toward VBHC, wherein different facets of the healthcare system prioritize aspects of value according to their strategic objectives and operational realities. Similar responses have occurred in international implementation of VBHC: the literature has indicated similar findings regarding variations in the conceptualization of VBHC among entities, as explained by the variations in policies established by the regulator and the payment models introduced by the funding entities (34). These variations reflect a dynamic and responsive healthcare system, but also highlight the need for a cohesive framework to unify these disparate efforts to achieve the common goal of value maximization.

VBHC Establishment

Regarding the second objective, our findings indicated that Saudi Arabia has demonstrated a commitment to the VBHC strategy, as supported by the analysis of three main government documents (25,26,31). These documents outline the strategic framework and plans for implementing VBHC in the country; however, the level of establishment remains not entirely clear, and progress reports from governmental entities appear to be limited. Considering the international experience, a global assessment conducted by The Economist in 2016 on the implementation of VBHC in 25 countries has highlighted the importance of high-level policies and government commitment in publishing strategies and plans to advance VBHC, which serve as a robust starting point for VBHC implementation (35). Moreover, the report has indicated that most countries have encountered prolonged timelines in aligning their healthcare systems with the components of VBHC, particularly given that this transition requires a paradigm shift. Consequently, our findings suggested that the careful and gradual progress made during the initial phases of transitional development is likely to contribute to expeditious use of VBHC.

Our findings revealed that Saudi Arabia has followed a strategic and deliberate approach to healthcare transformation through its initiatives in outcome measurement and financial reforms associated with the implementation of VBHC.

The efforts on outcome measurement are evidenced by the Saudi’s health sector, which focus on developing patient-reported outcome measures and patient-reported experience measures (PREMs), in a crucial step toward understanding and improving the value delivered to patients. The progress made in the PaRIS project, through a collaborative effort with the OECD, appears to be focused on the macro level, addressing regulations, tool validation, and the unification of reporting mechanisms. This finding aligns with the global experience: a review pertaining to several Western countries has highlighted the challenges in developing internationally comparable outcome sets, because of differences among countries. The authors of those study recommend that the first phase be dedicated to each country properly and correctly developing their own set of outcomes, emphasizing the importance of contextual considerations (36).

Regarding financial reforms, the shift toward value-based payment models, including introduction of bundled payments, formation of accountable care organizations, and application of value-based agreements, indicates a fundamental restructuring of the financial health model in Saudi Arabia to adhere to the value-based reimbursement of healthcare services. These financial reforms are critical in transitioning from a volume-based to a VBHC system (6). However, consideration of the local context, organization, and funding structure of the health system in each country is a major factor shaping the suitability of the chosen payment system (33,37). For example, fee-for-service-based systems, as in the US, differ from publicly run healthcare systems, as in the UK or other European countries, in their adaption of payment schemes while moving toward VBHC (33).

In the case of Saudi Arabia, the health system must continually analyze and tailor the underlying enablers and barriers to the implementation of VBHC to ensure the success of these financial reforms. Although the shift toward value-based payment models is a strategic step, the review highlights the need to carefully consider the contextual factors that might influence the effectiveness and sustainability of these reforms within the Saudi healthcare landscape.

Finally, although Saudi Arabia's commitment to VBHC is evident, the limited availability of progress reports and the known challenges faced by other countries in implementing VBHC prompt questions regarding the level of establishment and the pace of implementation in the Saudi context. Careful monitoring and transparent reporting of progress in aligning the healthcare system with VBHC principles will be crucial to assess the country's progress in transforming healthcare delivery.

Limitations

This scoping review has several limitations. First, because VBHC has only recently been introduced in Saudi Arabi, the database search yielded limited resources and scarce data; therefore, our findings were based on a limited number of articles. However, our findings are comparable to those from other studies conducted globally. Second, because scoping reviews are aimed at providing a broad overview of a research topic by mapping the existing literature, the primary focus of this review was not to undertake an in-depth analysis or critical appraisal of the included resources.

Implications for Policy and Practice

The findings of this review have substantial implications for policy and practice in Saudi Arabia. The alignment with global VBHC principles, coupled with efforts to tailor VBHC to the local context, provides a solid foundation for advancing healthcare quality and efficiency. However, to achieve the full potential of VBHC in Saudi Arabia, the following needs exist: first, strategic integration, which can be reflected by harmonizing the various VBHC initiatives and definitions into a cohesive national framework that clearly articulates the pathway toward value maximization; second, stakeholder engagement, through engaging a broad spectrum of stakeholders, including patients, providers, payers, policymakers, and researchers, in dialogue to foster a shared understanding and commitment to VBHC principles; and, finally, investment in building the infrastructure necessary for data collection, analysis, and reporting, to support robust outcome measurement and value assessment.

Implications for Future Research

Regarding future research directions, this review has identified critical areas for future research, including evaluation of VBHC impact (e.g., longitudinal studies assessing the effects of VBHC initiatives on healthcare outcomes, patient satisfaction, and cost-efficiency); best practices and lessons learned (e.g., comparative studies to identify best practices in VBHC implementation, both within Saudi Arabia and globally, to inform policy and practice); patient-centeredness (e.g., research focusing on the patient experience and engagement in VBHC, to understand how value is perceived and experienced by patients, and how it can be optimized); and, finally, policymaking (e.g., exploratory research to understand the roles of policymakers in accelerating the implementation of VBHC).

**Conclusion**

The transition toward value-based healthcare in Saudi Arabia is a promising but challenging journey. This scoping review described the current landscape of VBHC conceptualization and establishment, emphasizing the notable progress achieved and the challenges that lie ahead. As Saudi Arabia continues to navigate its healthcare transformation, the lessons learned and strategies used are expected to not only shape the future of healthcare in the Kingdom but also contribute to the regional and global discourse regarding value-based healthcare.

**Source of Funding**: The authors received no financial support for this research.

**Conflict of Interest:** The authors declare that they have no affiliations with, or involvement in, any organization or entity with any financial interest in the subject matter or materials discussed in this manuscript.

**Ethical Approval**: The study was approved by the Ethics Committee of Imam Abdulrahman bin Faisal University (IRB-PGS-2023-03-539) on November 28, 2023.

**Author Contributions:** B.H: Conceptualization, Methodology, Investigation, Writing—Original Draft, Project Administration. F.A: Conceptualization, Supervision. A.A: Conceptualization, Screening, Writing—Review & Editing, Supervision.

**Declaration of Generative AI and AI-assisted technologies in the writing process**

During the preparation of this work, the authors used PaperPal to enhance readability. After using this tool, the authors reviewed and edited the content as needed, and take full responsibility for the content of the publication.

**List of Abbreviations**

| VBHC | value-based healthcare |
| --- | --- |
| VIH | Center for Value in Health |
| PRISMA-ScR | Preferred Reporting Items for Systematic Reviews and Meta-Analyses extension for Scoping Reviews |
| MoH | MoH |
| PHA | Public Health Authority |
| CHI | Council of Health Insurance |
| HSTP | Health Sector Transformation Program |
| OECD | Organisation for Economic Co-operation and Development |
| NPHIES | National Platform for Health and Insurance Exchange Services |
| PaRIS | Patient Reported Indicator Survey |
| PREMs | patient-reported experience measures |

**R****eferences**

1. Al Asmri M, Almalki M, Fitzgerald G, Clark M. The public health care system and primary care services in Saudi Arabia: a system in transition. East Mediterr Health J. 2020 Apr 1;26(04):468–76.

2. Vision 2030. Vision 2030. [cited 2022 Oct 20]. Health Sector Transformation Program. Available from: https://www.vision2030.gov.sa/v2030/vrps/hstp

3. Kokko P. Improving the value of healthcare systems using the Triple Aim framework: A systematic literature review. Health Policy. 2022 Apr 1;126(4):302–9.

4. Renting N, Nutma E, Roemeling O, Smailhodzic E. Principles for the effective implementation of value-based healthcare: a scoping review and proposed process model for successful implementation [Internet]. In Review; 2022 Dec [cited 2023 Jun 11]. Available from: https://www.researchsquare.com/article/rs-2131671/v1

5. Sarkies MN, Francis-Auton E, Long JC, Partington A, Pomare C, Nguyen HM, et al. Implementing large-system, value-based healthcare initiatives: a realist study protocol for seven natural experiments. BMJ Open. 2020 Dec;10(12):e044049.

6. Porter ME, Teisberg EO. Redefining health care: creating value-based competition on results. Harvard business press; 2006.

7. NEJM Catalyst. What Is Value-Based Healthcare? Catal Carryover [Internet]. 2017 Jan [cited 2024 Jun 30];3(1). Available from: https://catalyst.nejm.org/doi/full/10.1056/cat.17.0558

8. Gray M. Value based healthcare. BMJ. 2017 Jan 27;j437.

9. Koff E, Lyons N. Implementing value‐based health care at scale: the NSW experience. Med J Aust. 2020 Feb;212(3):104.

10. Center for Improving Value in Health Saudi Arabia. Transformation Towards Sustainable Health Systems Through Value Based Health Care [Internet]. Riyadh.: Global Innovation Hub for Improving Value in Health; 2020 [cited 2024 Jun 26]. Available from: chrome-extension://efaidnbmnnnibpcajpcglclefindmkaj/https://www.g20hub.org/files/20201019_GIH_Country-Case-Studies-Paper_For-Publication_v2.0.pdf

11. Govender T, Al Bunyan, Anidi E. Drivers and Barriers for implementation of Value-based healthcare in Middle East and North Africa (MENA) region. The Global Innovation Hub for Improving Value in Health; 2021.

12. Value in Health. Definition of value in health in Saudi Arabia. 2020.

13. Value in Health. A Framework for Measuring Health System Value: Strengthening the measurement of outcomes and costs in the Saudi health system. The Center for Value in Health; 2022.

14. Munn Z, Peters MDJ, Stern C, Tufanaru C, McArthur A, Aromataris E. Systematic review or scoping review? Guidance for authors when choosing between a systematic or scoping review approach. BMC Med Res Methodol. 2018 Dec;18(1):143.

15. Arksey H, O’Malley L. Scoping studies: towards a methodological framework. Int J Soc Res Methodol. 2005 Feb;8(1):19–32.

16. Tricco AC, Lillie E, Zarin W, O’Brien KK, Colquhoun H, Levac D, et al. PRISMA Extension for Scoping Reviews (PRISMA-ScR): Checklist and Explanation. Ann Intern Med. 2018 Oct 2;169(7):467–73.

17. Haddaway NR, Grainger MJ, Gray CT. citationchaser: An R package and Shiny app for forward and backward citations chasing in academic searching [Internet]. Zenodo; 2021 [cited 2024 Feb 25]. Available from: https://zenodo.org/record/4543513

18. Clark J, Glasziou P, Del Mar C, Bannach-Brown A, Stehlik P, Scott AM. A full systematic review was completed in 2 weeks using automation tools: a case study. J Clin Epidemiol. 2020 May;121:81–90.

19. Abdalla R, Pavlova M, Groot W. Association of patient experience and the quality of hospital care. Int J Qual Health Care J Int Soc Qual Health Care. 2023 Jul 5;35(3).

20. Abu-Shraie N, Alhammad A, Balkhi B, Al-Jedai A. Implementation of risk-sharing agreements in Saudi Arabia: Comparison and reflection on the NICE Model. Trop J Pharm Res. 2023 May;22(5):1121–31.

21. Alkhudair N, Alshamrani M, Samarkandi H, Almodaheem H, Alabdulkarim H, Alsaqaaby M, et al. Cancer Management in Saudi Arabia: Recommendations by the Saudi Oncology HeAlth Economics ExpeRt GrouP (SHARP). Saudi Pharm J SPJ Off Publ Saudi Pharm Soc. 2021 Feb;29(2):115–20.

22. Memish ZA, Alqahtani AS, Al-Azemi N, Abu Alhamayel N, Saeedi M, Abuzinada S, et al. A New Era of National Guideline Development in Saudi Arabia. J Epidemiol Glob Health. 2022 Dec;12(4):373–9.

23. Abdalla R, Pavlova M, Groot W. Prioritizing Outcome Measures for Value-Based Healthcare: Physicians’ Perspectives in Saudi Arabia. Value Health Reg Issues. 2023 Sep;37:62–70.

24. Al-Ghamdi M, AlTamimi M, Al-Azmi N, Al-Ghaith T, AlMuaither F, AlThunayyan R, et al. Development of national framework for health status and health system performance indicators in Saudi Arabia. J Infect Public Health. 2023 Feb 1;16(2):295–302.

25. MoH. Health Sector Transformation Strategy. 2018.

26. HSTP. Health Sector Transformation Program Delivery Plan. 2021.

27. Value in Health. 2020 Annual report. The Center for Improving Value in Health; 2020.

28. Value in Health. 2021 Annual Report. The Center for Improving Value in Health; 2021.

29. Reka H, Almaghrabi A, Alghamdi DS. White Paper on Value-Based Payment. Council of Health Insurance; 2022.

30. Garcia J, Reddy C, Atun R. Transitioning to High-Value Health Systems in G20+ Countries. Health Systems Innovation Lab: Harvard University; 2023.

31. Value in Health. Definition of Value in Health in Saudi Arabia. Value in Health Center; 2020.

32. Hurst L, Mahtani K, Pluddemann A, Lewis S, Harvey K, Briggs A, et al. Defining Value-based Healthcare in the NHS. Cent Evid-Based Med Rep 201904 [Internet]. 2019 [cited 2023 Mar 24];(4). Available from: https://ora.ox.ac.uk/objects/uuid:2ab0a667-2a14-4242-a30f-1bdbeaad95ef

33. Mjåset C, Ikram U, Nagra NS, Feeley TW. Value-Based Health Care in Four Different Health Care Systems. Catal Non-Issue Content [Internet]. 2020 Nov 10 [cited 2023 Sep 22];1(6). Available from: https://catalyst.nejm.org/doi/full/10.1056/CAT.20.0530

34. Staalduinen DJ van, Bekerom P van den, Groeneveld S, Kidanemariam M, Stiggelbout AM, Marle ME van den A van. The implementation of value-based healthcare: a scoping review. BMC Health Serv Res. 2022 Mar 1;22(1):1–8.

35. The Economist Intelligence Unit. Value-based healthcare: A global assessment [Internet]. The Economist; 2016. Available from: https://impact.economist.com/perspectives/sites/default/files/EIU_Medtronic_Findings-and-Methodology_1.pdf

36. Eijsink JFH, Fabian AM, Vervoort JPM, Al Khayat MNMT, Boersma C, Postma MJ. Value-based health care in Western countries: a scoping review on the implementation of patient-reported-outcomes sets for hospital-based interventions. Expert Rev Pharmacoecon Outcomes Res. 2023 Jan 2;23(1):1–13.

37. Wheeler SB, Spencer J, Rotter J. Toward Value in Health Care: Perspectives, Priorities, and Policy. N C Med J. 2018;79(1):62–5.

Tables

Table 1: Database search syntax

| PubMed | (“Value Based Health Insurance”[Mesh] OR “value based care”[All Fields] OR “value based healthcare”[All Fields] OR “value based healthcare”[All Fields] OR “VBHC”[All Fields]) AND (“Saudi Arabia”[Mesh] OR “Saudi Arabia”[All Fields]) |
| --- | --- |
| Scopus | TS=(“Value-Based Health Insurance” OR “value-based care” OR “VBHC” OR “value-based healthcare” OR “value based care” OR “value based healthcare” OR “value based healthcare” OR “VBHC”) AND CU=(“Saudi Arabia”) |
| Web of Science | TITLE-ABS-KEY ("Value-Based Health Insurance" OR "value-based care" OR "VBHC" OR "value-based healthcare" OR "value based care" OR "value based healthcare" OR "value based healthcare" OR "VBHC") AND AFFILCOUNTRY ( "Saudi Arabia") |

Table 2: Definition overview for included articles

| Evidence Information | | | | VBHC Conceptualization |
| --- | --- | --- | --- | --- |
|  | **Reference** | **Study Type** | **Aim** | **VBHC Definition** |
| 1 | (19) | Retrospective observational study | The association between clinical outcomes and PREMs in hospitals in Saudi Arabia was assessed to inform VBHC reforms. | "Value-based healthcare (VBHC) aims to achieve the **best health outcomes at the lowest cost** to optimize value for patients" p. 1 |
| 2 | (21) | Focus groups | A group of experts convened to discuss key factors affecting the current state of cancer management in Saudi Arabia and to agree on a list of recommendations, with a focus on value-based care, considering evidence, patients, and costs. | VBHC is aimed at "balancing the clinical benefits versus costs when making decisions related to drug reimbursement" p. 116 |
| 3 | (24) | Mixed methods | The aim was to provide an overview of a new, unified national framework. | The article describes the elements (goals) of VBHC by stating the need to measure value, outcomes, and cost, but its focus is on outcome measurement. p. 296 |
| 4 | (20) | Review | This study was aimed at describing the status of **risk-sharing agreement**s, trends in utilization of RSAs, and challenges in RSA implementation in Saudi Arabia. | The article describes "increasing the quality of care and quality of life of patients while utilizing resources more efficiently." p.1126 |
| 5 | (23) | Cross-sectional | This study was aimed at determining the top priority outcomes that must be measured for all patients, regardless of the medical condition, from the perspective of physicians. | "Value-based healthcare (VBHC) implies a focus on what patients value most." p. 62 |
| 6 | (22) | Progress report | The aim was to develop a national clinical guideline. | Value was linked to cost only. A major solution to develop a national guideline is the "systematic consideration of cost as one of the contextual factors" in focusing on local needs and values. p. 376 |

Table 3: Definition Overview of Included Governmental Documents and Other Reports

| **Evidence Information** | | | | **VBHC Conceptualization** |
| --- | --- | --- | --- | --- |
| **Type** | **Entity** | **Reference** | **Evidence Type** | **VBHC Definition** |
| Governmental document | MoH | (25) | HSTP strategy delivery report | "Outcome divided by cost” p.59 |
| Governmental document | MoH | (26) | HTP strategy delivery plan | No definition, but describes the elements of VBHC throughout the document |
| Governmental document | VIH | (31) | Policy perspective | "Achieving the best health outcomes with the optimal and fair allocation and best utilization of resources where ‘outcomes’ relate to benefits delivered for individuals, communities and the population; and, ‘resources’ include all human, capital and natural resources" p. 4 |
| Governmental document | VIH | (27) | Annual report | "Achieving the best health outcomes with the optimal and fair allocation and best utilization of resources where ‘outcomes’ relate to benefits delivered for individuals, communities and the population; and, ‘resources’ include all human, capital and natural resources" p. 14 |
| Governmental document | VIH | (28) | Annual report | No clear definition. but elements of VBHC are described throughout the document |
| Governmental document | CHI | (29) | White paper | “The quality of patient outcomes relative to the dollars expended" p. 5 |
| Report | GIH | (11) | Report | “Aims to improve the health outcomes of people while optimizing health systems to reduce healthcare costs " p. 12 |
| Report | GIH | (30) | Report | “High-value health service as any health service at the population level or individual level delivered effectively, efficiently, responsively, and equitably in a health system” p.14 |

Table 4: Overview of the Establishment Levels on Outcome Measurement

| **Evidence Information** | | **Establishment Level** | | |
| --- | --- | --- | --- | --- |
|  |  | **Outcome** | | |
| **Type** | **Reference** | **Individuals** | **Communities** | **Population** |
| Article | (19) | "The Saudi Patient Experience Centre has established the Patient Experience Measurement programme with different outcomes that mainly include developing, comparing, and improving patient experience at the national and international levels. Yet association between PREMS and clinical outcomes was not tested." p. 1–2 | | |
| Article | (24) | "The framework has been designed in such a way that, in the long-term, it could be further stratified by population groups (condition specific) to provide a better view of value and equity, as resources and outcomes differ across different conditions. Condition-specific indicators could be considered across the Health System framework, especially but not limited to the ‘outcomes’ subdomain, as per the following. These would, allow for the measurement of value (considering allocated costs, resources, outcomes, and experiences) at the level of specific conditions." p. 301 | | |
| Article | (23) | "The transition to VBHC is still in its early stages and clinician involvement in developing outcome measures is limited. This research is done as an initial step towards the implementation of disease-specific outcome sets." p. 68 | | |
| Article | (22) | "Based on a data-driven impact/effort analysis, selection of the initial 12 guideline" p. 375. These guidelines will be nationally adopted reliable sources for clinicians. | | |
| Government document | (27) | “The Center also initiated a multi-year research program in collaboration with OECD to measure patient-reported outcomes and experiences in primary health care (PaRIS project).” p. 18 | | |
| Government document | (28) | “The Center for value in health is taking a country-wide lead in the delivery of this primary survey in PHCs primarily to measure patient reported outcomes and experiences in order to identify the potential areas for improvement in care provision and to maintain focus on achieving improved value.” p. 13 | | |
| Report | (30) | "Various demonstration projects are being implemented to measure patient experiences, including the OECD's PaRIS project on measuring patient-reported outcomes and patient-reported experience in patients with chronic diseases." p. 55 | | |

Table 5: Overview of the Establishment Levels in Resource Allocation

| **Evidence Information** | | **VBHC Establishment Level** |
| --- | --- | --- |
|  |  | **Resources** |
| **Type** | **Reference** | **Allocation (Financial Reforms)** |
| Article | (21) | "In 2019, MOH established 5 value-based agreements with pharmaceutical manufacturers." Value-based agreements "are type of reimbursement scheme between payers and medical product manufacturers that have proven to be a useful tool in addressing increasing cost pressures, uncertain effectiveness, and ensuring value in healthcare decision-making." p. 118. |
| Article | (20) | "Saudi Arabia did implement several RSAs between 2018 and 2020. However, in KSA, the experience with RSAs is a recent phenomenon that is still in the early phase. Therefore, there is need for future assessment of RSAs with respect to success rate in achieving expected objectives." p. 1127 |
| Government document | (29) | The following financial reforms are in place:  NPHIES  Patient experience measurement (customer satisfaction survey PG)  Classification system for HC services  Provider classification  Saudi billing system |
| Report | (30) | "The government is also developing value-based procurement initiatives, such as Nupco's implementation of value-based purchasing models for most drugs for public hospitals." p. 55 |

Table 6: Overview of the Establishment Levels in Resource Utilization

| **Evidence Information** | | **VBHC Establishment Level** |
| --- | --- | --- |
|  |  | **Resources** |
| **Type** | **Reference** | **Utilization (Provider Reforms)** |
| Government  document | (27) | “In 2020, the Center ran a physician engagement project to inform this topic, and one of the outputs of this project was a preliminary knowledge, competency and behavioural framework to support the delivery of value.” p. 19 |

**Appendix A**

**Preferred Reporting Items for Systematic reviews and Meta-Analyses extension for Scoping Reviews (PRISMA-ScR) Checklist**

| **SECTION** | **ITEM** | **PRISMA-ScR CHECKLIST ITEM** | **REPORTED ON PAGE #** |
| --- | --- | --- | --- |
| **TITLE** | | | |
| Title | 1 | Identify the report as a scoping review. | 1 |
| **ABSTRACT** | | | |
| Structured summary | 2 | Provide a structured summary that includes (as applicable): background, objectives, eligibility criteria, sources of evidence, charting methods, results, and conclusions that relate to the review questions and objectives. | 1 |
| **INTRODUCTION** | | | |
| Rationale | 3 | Describe the rationale for the review in the context of what is already known.    Explain why the review questions/objectives lend themselves to a scoping review approach. | 2–4  5 |
| Objectives | 4 | Provide an explicit statement of the questions and objectives being addressed with reference to their key elements (e.g., population or participants, concepts, and context) or other relevant key elements used to conceptualize the review questions and/or objectives. | 3 |
| **METHODS** | | | |
| Protocol and registration | 5 | Indicate whether a review protocol exists; state if and where it can be accessed (e.g., a Web address); and, if available, provide registration information, including the registration number. | NA |
| Eligibility criteria | 6 | Specify characteristics of the sources of evidence used as eligibility criteria (e.g., years considered, language, and publication status), and provide a rationale. | 6 |
| Information sources* | 7 | Describe all information sources in the search (e.g., databases with dates of coverage and contact with authors to identify additional sources), as well as the date the most recent search was executed. | 6 |
| Search | 8 | Present the full electronic search strategy for at least one database, including any limits used, such that it could be repeated. | 24 |
| Selection of sources of evidence† | 9 | State the process for selecting sources of evidence (i.e., screening and eligibility) included in the scoping review. | 6 |
| Data charting process‡ | 10 | Describe the methods of charting data from the included sources of evidence (e.g., calibrated forms or forms that have been tested by the team before their use, and whether data charting was done independently or in duplicate) and any processes for obtaining and confirming data from investigators. | 7–8 |
| Data items | 11 | List and define all variables for which data were sought and any assumptions and simplifications made. | NA |
| Critical appraisal of individual sources of evidence§ | 12 | If done, provide a rationale for conducting a critical appraisal of included sources of evidence; describe the methods used and how this information was used in any data synthesis (if appropriate). | NA |
| Synthesis of results | 13 | Describe the methods of handling and summarizing the data that were charted. | 7–8 |
| **RESULTS** | | | |
| Selection of sources of evidence | 14 | Give numbers of sources of evidence screened, assessed for eligibility, and included in the review, with reasons for exclusions at each stage, ideally using a flow diagram. | 7 |
| Characteristics of sources of evidence | 15 | For each source of evidence, present characteristics for which data were charted and provide the citations. | 25–29 |
| Critical appraisal within sources of evidence | 16 | If done, present data on critical appraisal of included sources of evidence (see item 12). | NA |
| Results of individual sources of evidence | 17 | For each included source of evidence, present the relevant data that were charted that relate to the review questions and objectives. | 25–29 |
| Synthesis of results | 18 | Summarize and/or present the charting results as they relate to the review questions and objectives. | 8–13 |
| **DISCUSSION** | | | |
| Summary of evidence | 19 | Summarize the main results (including an overview of concepts, themes, and types of evidence available), link to the review questions and objectives, and consider the relevance to key groups. | 14–17 |
| Limitations | 20 | Discuss the limitations of the scoping review process. | 17 |
| Conclusions | 21 | Provide a general interpretation of the results with respect to the review questions and objectives, as well as potential implications and/or next steps. | 19 |
| **FUNDING** | | | |
| Funding | 22 | Describe sources of funding for the included sources of evidence, as well as sources of funding for the scoping review. Describe the role of the funders of the scoping review. | 19 |
| **SECTION** | **ITEM** | **PRISMA-ScR CHECKLIST ITEM** | **REPORTED ON PAGE #** |
| **TITLE** | | | |
| Title | 1 | Identify the report as a scoping review. | 1 |
| **ABSTRACT** | | | |
| Structured summary | 2 | Provide a structured summary that includes (as applicable): background, objectives, eligibility criteria, sources of evidence, charting methods, results, and conclusions that relate to the review questions and objectives. | 1–2 |
| **INTRODUCTION** | | | |
| Rationale | 3 | Describe the rationale for the review in the context of what is already known.    Explain why the review questions/objectives lend themselves to a scoping review approach. | 2–4  6 |
| Objectives | 4 | Provide an explicit statement of the questions and objectives being addressed with reference to their key elements (e.g., population or participants, concepts, and context) or other relevant key elements used to conceptualize the review questions and/or objectives. | 4 |
| **METHODS** | | | |
| Protocol and registration | 5 | Indicate whether a review protocol exists; state if and where it can be accessed (e.g., a Web address); and, if available, provide registration information, including the registration number. | NA |
| Eligibility criteria | 6 | Specify characteristics of the sources of evidence used as eligibility criteria (e.g., years considered, language, and publication status), and provide a rationale. | 7 |
| Information sources* | 7 | Describe all information sources in the search (e.g., databases with dates of coverage and contact with authors to identify additional sources), as well as the date the most recent search was executed. | 7 |
| Search | 8 | Present the full electronic search strategy for at least one database, including any limits used, such that it could be repeated. | 25 |
| Selection of sources of evidence† | 9 | State the process for selecting sources of evidence (i.e., screening and eligibility) included in the scoping review. | 7 |
| Data charting process‡ | 10 | Describe the methods of charting data from the included sources of evidence (e.g., calibrated forms or forms that have been tested by the team before their use, and whether data charting was done independently or in duplicate) and any processes for obtaining and confirming data from investigators. | 8–9 |
| Data items | 11 | List and define all variables for which data were sought and any assumptions and simplifications made. | NA |
| Critical appraisal of individual sources of evidence§ | 12 | If done, provide a rationale for conducting a critical appraisal of included sources of evidence; describe the methods used and how this information was used in any data synthesis (if appropriate). | NA |
| Synthesis of results | 13 | Describe the methods of handling and summarizing the data that were charted. | 8–9 |
| **RESULTS** | | | |
| Selection of sources of evidence | 14 | Give numbers of sources of evidence screened, assessed for eligibility, and included in the review, with reasons for exclusions at each stage, ideally using a flow diagram. | 8 |
| Characteristics of sources of evidence | 15 | For each source of evidence, present characteristics for which data were charted and provide the citations. | 25–29 |
| Critical appraisal within sources of evidence | 16 | If done, present data on critical appraisal of included sources of evidence (see item 12). | NA |
| Results of individual sources of evidence | 17 | For each included source of evidence, present the relevant data that were charted that relate to the review questions and objectives. | 25–29 |
| Synthesis of results | 18 | Summarize and/or present the charting results as they relate to the review questions and objectives. | 9–14 |
| **DISCUSSION** | | | |
| Summary of evidence | 19 | Summarize the main results (including an overview of concepts, themes, and types of evidence available), link to the review questions and objectives, and consider the relevance to key groups. | 25–29 |
| Limitations | 20 | Discuss the limitations of the scoping review process. | 17 |
| Conclusions | 21 | Provide a general interpretation of the results with respect to the review questions and objectives, as well as potential implications and/or next steps. | 18 |
| **FUNDING** | | | |
| Funding | 22 | Describe sources of funding for the included sources of evidence, as well as sources of funding for the scoping review. Describe the role of the funders of the scoping review. | 19 |

JBI = Joanna Briggs Institute; PRISMA-ScR = Preferred Reporting Items for Systematic reviews and Meta-Analyses extension for Scoping Reviews.

* Locations from which *sources of evidence* (see second footnote) are compiled, such as bibliographic databases, social media platforms, and Web sites.

† A more inclusive/heterogeneous term used to account for the different types of evidence or data sources (e.g., quantitative and/or qualitative research, expert opinion, and policy documents) that may be eligible in a scoping review as opposed to only studies. This is not to be confused with *information sources* (see first footnote).

‡ The frameworks by Arksey and O’Malley (6) and Levac and colleagues (7) and the JBI guidance (4, 5) refer to the process of data extraction in a scoping review as data charting*.*

§ The process of systematically examining research evidence to assess its validity, results, and relevance before using it to inform a decision. This term is used for items 12 and 16 instead of "risk of bias" (which is more applicable to systematic reviews of interventions) to include and acknowledge the various sources of evidence that may be used in a scoping review (e.g., quantitative and/or qualitative research, expert opinion, and policy document).

*From:* Tricco AC, Lillie E, Zarin W, O'Brien KK, Colquhoun H, Levac D, et al. PRISMA Extension for Scoping Reviews (PRISMAScR): Checklist and Explanation. Ann Intern Med. 2018;169:467–473. [doi: 10.7326/M18-0850](http://annals.org/aim/fullarticle/2700389/prisma-extension-scoping-reviews-prisma-scr-checklist-explanation).
